# The response to Typhi Vi vaccination is compromised in individuals with primary immunodeficiency

**DOI:** 10.1016/j.heliyon.2017.e00333

**Published:** 2017-06-23

**Authors:** Jeevani Kumarage, Suranjith L. Seneviratne, Vijitha Senaratne, Amitha Fernando, Kirthi Gunasekera, Bandu Gunasena, Padmalal Gurugama, Sudath Peiris, Antony R. Parker, Stephen Harding, Nilhan Rajiva de Silva

**Affiliations:** a Department of Immunology, Medical Research Institute, Colombo 8, Sri Lanka; b Institute of Immunity and Transplantation, Royal Free Hospital, London, UK; c Department of Surgery, Faculty of Medicine, University of Colombo, Sri Lanka; d National Hospital for Respiratory Diseases, Walisara, Sri Lanka; e National Hospital of Sri Lanka/Central Chest Clinic, Colombo, Sri Lanka; f Department of Clinical Biochemistry and Immunology, Cambridge University Hospitals NHS Foundation Trust, Hills road, Cambridge, UK; g Epidemiology Unit, Ministry of Health, Sri Lanka; h The Binding Site Group Limited, 8 Calthorpe Road, Edgbaston, Birmingham, B15 1QT, UK

**Keywords:** Health sciences, Biological sciences, Infectious disease, Vaccines, Immunology, Medicine, Pediatrics

## Abstract

Measurement of an individuals ability to respond to polysaccharide antigens is a crucial test to determine adaptive immunity. Currently the response to Pneumovax^®^ is utilized but with the success of Prevnar^®^, measurement of the response to Pneumovax may be challenging. The aim of the study was to assess the response to Typhi Vi vaccination in both children and adult control groups and patients with primary immunodeficiency (PID). In the control groups, >95% of the individuals had pre Typhi Vi vaccination concentrations <100 U/mL and there was significant increase in concentration post Typhi Vi vaccination (p<0.0001) with>94% achieving ≥3 fold increase in concentration (FI). The response to Typhi Vi vaccination was significantly lower in both children (*p* = 0.006) and adult (*p* = 0.002) PID groups when compared to their control groups. 11% and 55% of the children and adult PID groups respectively did not obtain a response >3FI. There were no significant differences between the responses obtained in the children and adult PID groups. When all individuals with PID were separated into those with either hypogammaglobulinemia (HYPO) or common variable immunodeficiency (CVID), both groups had a significantly lower median FI than the control group (19, 95%CI 5–56 vs 59, 95%CI 7–237; *p* = 0.01 and 1, 95%CI 1–56 vs 32, 95%CI 5–136; *p* = 0.005). Further, a >3FI differentiated the antibody responses between both the CVID and HYPO groups and their control groups (AUC: 0.83, 95%CI: 0.65–1.00, *p* = 0.005 and 0.81, 95% CI: 0.65–0.97, *p* = 0.01). The data suggests that measurement of the response to Typhi Vi vaccination could represent a complementary assay for the assessment of the response to a polysaccharide vaccine.

## 1 Introduction

Defective production of specific antibodies in response to polysaccharide antigens is a major risk for infection and other complications in patients with antibody deficiencies. Currently, specific antibody responses to Pneumovax are measured in individuals with symptoms suggestive of a deficiency of antibody production [1]. Interpretation of the response to pneumococcal vaccination is becoming more challenging. High pneumococcal pre-immunization levels in the general population may limit the response to vaccination [2]. Cross-reacting antibodies [3] and different immunogenicities of the large number of different serotypes may complicate interpretation of the response further. The undoubted success of the polysaccharide-protein conjugated vaccine Prevnar may hide the response to the pure polysaccharide vaccine and with the initial reports of a polysaccharide-protein conjugated vaccine developed for 15 serotypes, availability of Pneumovax may become limited [4].

Protection against *Salmonella* Typhoid fever currently involves the immunization of at risk populations living in areas with endemic *Salmonella* fever and of visitors to such regions with a polysaccharide vaccine. The vaccines are targeted to the capsular polysaccharide Vi antigen [5]. Measurement of Typhi Vi antibodies may be a suitable additional candidate for the assessment of the response to polysaccharide antigens because (1) interpretation is less complicated due to lack of multiple serotypic components and thus less cross reactivity, (2) there is no conjugated polysaccharide vaccine currently in routine use globally, and (3) the pre Typhi Vi vaccination concentrations should be generally low in most populations [6, 7, 8].

It has been reported that 95% of healthy volunteers vaccinated with Typhim Vi achieved a >3 fold increase (FI) in the concentration of Typhim Vi antibodies between pre and post vaccination [6]. Sanchez-Ramon et al. recently reported that a 3FI could aid differentiation of patients with common variable immunodeficiency (CVID) from healthy volunteers and those with hypogammaglobulinemia (HYPO) [8].

In the present study, we compared the response to Typhi Vi vaccination in both children and adult control groups to that measured in patients with primary immunodeficiencies (PID). In addition we compared the responses between individuals with CVID or HYPO.

## 2 Materials and methods

### 2.1 Control and patient populations

Patients who were referred to the Department of Immunology, Medical Research Institute (MRI), Colombo, Sri Lanka for routine immunological evaluation between August 2011 and December 2013, were recruited to the study.

For all participants, a blood sample was drawn for baseline pre Typhi Vi vaccination antibody concentrations. All individuals were vaccinated with Typbar^®^ (Barat Biotech, India). Approximately 28 days post Typhi Vi vaccination, blood was drawn from all subjects for post vaccination analysis. Assessment of Typhi Vi vaccination did not form part of the routine immunological evaluation. All samples were stored at −80 °C.

These patients, all of whom presented with recurrent infections, were divided into the following groups:

1. Control group for adults (n = 24).
2. Control group for children (n = 20).
3. Adult PID group (1HYPO and 8CVID patients; n = 9).
4. Children PID group (8HYPO and 1CVID patients; n = 9).

The demographics and characteristics of each group are shown in Table 1.

**Table 1:** Demographics and clinical characteristics of all groups used in this study.

	Adult control group	Adult PID group	*P* value	Children control group	Children PID group	*P* value
Number of patients	24	9	–	20	9	–
Gender (M:F)	7:17	5:4	NS	9:11	5:4	NS
Age years (median, range)	38 (18–54)	30 (22–50)	NS	7 (5–16)	6 (5–12)	NS
IgG (median, range; g/L)	9.8 (6.0–16.4)	3.0 (0.04–5.1)	<0.0001	10.8 (7.1–18.2)	5.1 (3.3–5.6)	<0.0001
IgA (median, range; g/L)	2.6 (0.7–3.7)	0.16 (0.06–3.2)	0.0005	1.3 (0.45–3.5)	0.5 (0.08–0.91)	0.0012
IgM (median, range; g/L)	1.1 (0.5–2.8)	0.41 (0.016–1.5)	0.002	1.1 (0.55–2.5)	0.5 (0.18–1.2)	0.001

NS means not significant (*p* > 0.05).

Both CVID and HYPO patients were diagnosed according to the European Society of Immunodeficiencies (ESID) and the Pan American Group for Immune deficiency (PAGID) criteria [9, 10, 11]. CVID was defined as a male or female patient with a marked decrease (at least 2 SD below the mean for age) in serum IgG and IgA, onset of immunodeficiency at greater than 2 years of age, absent isohemagglutinins and/or poor response to vaccines with the exclusion of defined causes of hypogammaglobulinemia.

All samples were taken before administration of any antibody replacement therapy.

### 2.2 Ethics approval

Informed written consent was obtained from all participants and studies were performed in accordance with the Declaration of Helsinki. All procedures were approved by the local ethics committee (Ethics Review Committee of the Medical Research Institute, Colombo).

### 2.3 Measurement of Typhi Vi IgG antibodies

Anti Typhi Vi IgG were measured using the VaccZyme *Salmonella* Typhi Vi IgG ELISA (The Binding Site Group, Birmingham, UK). The assay was run according to manufacturer’s instructions. The measuring range of the assay was 7.4–600 U/mL. Fold increase in concentration (FI) was calculated using the following formula: Post vaccination concentration/pre vaccination concentration. For the purpose of statistical analysis, all values <7.4 U/mL were given a value of 7.4 U/mL and as a consequence all FI are represented as “at least” the FI achieved. FI in concentration was assessed using a cutoff of 3 [6, 8]. Responders were defined as individuals obtaining a FI > 3 and non responders <3. The term “ concentration” refers to the concentration of Typhi Vi IgG antibodies.

### 2.4 Statistical analysis

Shapiro-Wilks, Mann Whitney U, Wilcoxon tests and ROC analysis were performed using Prism Graphics Program. A *p* < 0.05 was considered statistically significant.

## 3 Results

### 3.1 Clinical characteristics of control and patient groups

Table 1 show the demographics and characteristics of the groups used in this study. The ages and genders were not significantly different between the control groups and the PID groups. Serum IgG, IgA and IgM concentrations were significantly lower for both PID groups compared to their appropriate control groups.

### 3.2 Typhi Vi response in children and adult control groups

**Fig. 1:**
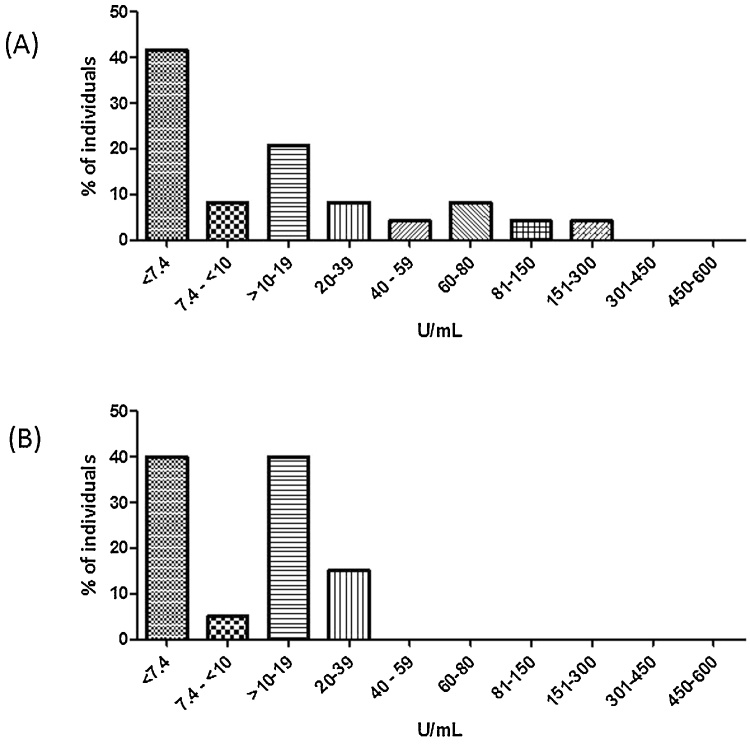
Baseline Typhi Vi IgG concentrations in children and adult control groups. The Typhi Vi pre vaccination concentrations were determined in the two control groups and separated into concentration ranges. The % individuals in the different concentration ranges were calculated. (A) Adult control group (n = 24) and (B) Children control group (n = 20).

The median pre Typhi Vi vaccination concentrations are shown in Table 2. The pre Typhi Vi vaccination concentrations were divided into concentration ranges and the percentage of individuals in those ranges calculated (Fig. 1). In both control groups, >95% of the individuals had Typhim Vi concentrations <100 U/mL with the exception of 2 individuals in the adult control group (119 U/mL and 270 U/mL).

**Table 2:** Characteristics of the Typhi Vi responses.

Responses	Adult control group	Adult PID group	*P* value	Children control group	Children PID group	*P* value
Pre vaccination (median 95% CI, U/mL)	10 (7.4–232)	7.4 (7.4–100)	NS	11 (7.4–28)	9 (7.4–179)	NS
Post vaccination (median 95% CI, U/mL)	519 (80–2779)	11 (7.4–1746)	0.03	679 (60–3029)	219 (12–815)	0.009
FI (median 95% CI, U/mL)	32 (5–135)	2 (1–56)	0.002	59 (7–236)	18 (1–56)	0.006

NS means not significant (*p* > 0.05).

**Fig. 2:**
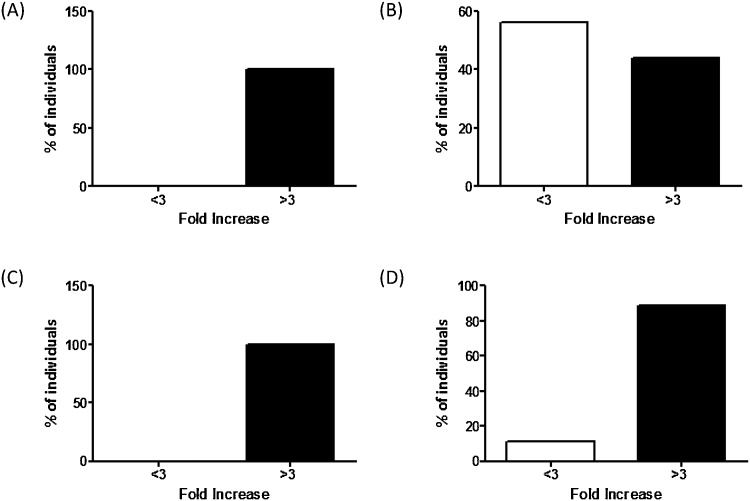
Responses to Typhi Vi vaccination in control groups and PID groups. Typhi Vi responses were divided into <3 or>3 FI and represented as a percentage for (A) Adult control group, (B) Adult PID group, (C) Children control group and (D) Children PID group.

The response to Typhi Vi vaccination is shown in Fig. 2 and Table 2. In the two control groups there was significant increase in Typhi Vi concentration post vaccination (*p* < 0.0001) with>94% achieving ≥3 FI (Fig. 2A and C).

### 3.3 Typhi Vi response in children and adult primary immunodeficiency groups

There were no significant differences between the pre Typhi Vi vaccination concentrations in either PID groups and the appropriate control group (*P* = 0.1–0.5, Table 2). The response to Typhi Vi vaccination was significantly lower in both the children and adult PID groups when compared to their control groups (Fig. 2B and D and Table 2).

11% and 55% of the children and adult PID groups respectively did not obtain a response >3 FI and of the individuals with >3 FI, 25% of each PID group had post Typhi Vi vaccination concentrations lower that the lower limit of the respective normal ranges (<80 U/mL and <60 U/mL respectively, Table 2).

### 3.4 Comparison of children vs adult responses in the control groups

The maximum pre Typhi Vi vaccination concentration was lower in children than adults (<39 U/mL vs <300 U/mL, Fig. 1) but there were no significant differences between the median pre Typhi Vi or post Typhi Vi vaccination concentrations (*p*> 0.3). The FI was higher in children compared to adults but did not reach statistical significance (*p* = 0.06).

### 3.5 Comparison of responses for CVID and HYPO

**Fig. 3:**
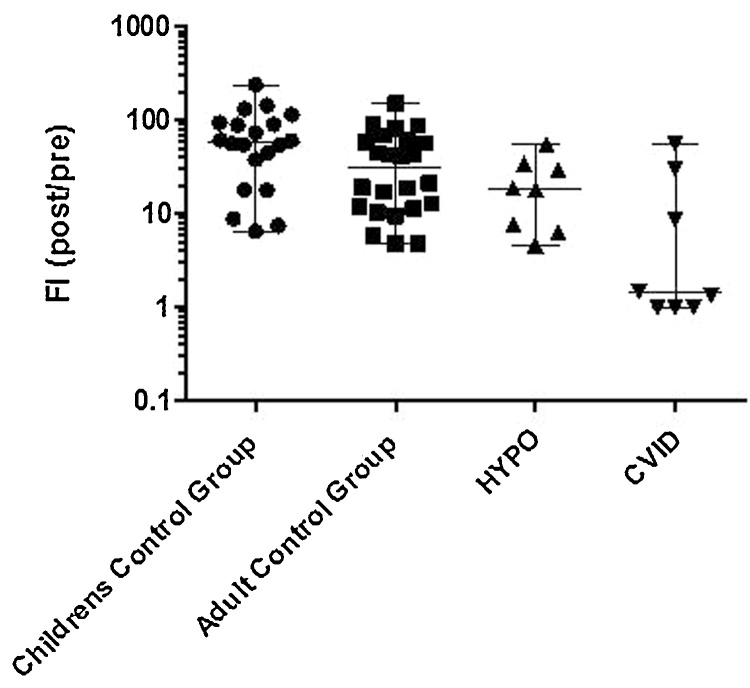
Responses to Typhi Vi vaccination in control groups, HYPO and CVID patients. Typhi Vi responses were assessed in the Children control group (n = 20), Adult control group (n = 24), HYPO (n = 8) and CVID (n = 8) groups.

When the PID patients were divided into those with HYPO and those with CVID, both groups had a significantly lower median FI post Typhi Vi vaccination than the control groups (19, 95% CI 5–56 vs 59, 95% CI 7–236; *p* = 0.01 and 1, 95% CI 1–56 vs 32, 95%CI 5–135; *p* = 0.005 respectively, Fig. 3). A 3 FI aided differentiation of the antibody responses between the CVID and HYPO groups and their control groups (AUC: 0.83, 95% CI: 0.65–1.00, *p* = 0.005 and 0.81, 95% CI: 0.65–0.97, *p* = 0.01 respectively).

## 4 Discussion

The failure to respond to a polysaccharide vaccine is a reflection of a defective adaptive immune system in response to polysaccharide antigens and may leave an individual at risk of recurrent infections. The IgG response to Pneumovax is currently used to assess the ability to produce polysaccharide antigen antibodies but this may have shortcomings. The IgG response to Typhi Vi vaccination may represent an additional tool to assess the response to a polysaccharide antigen.

The Typhi Vi pre vaccination concentrations were very low in the two control groups with >95% possessing concentrations <100 U/mL. This is in agreement with previous reports [6, 7, 8] and suggests that the pre vaccination concentration of Typhi Vi antibodies may not be a inhibiting factor for achieving a>3 FI as has been observed for the response to Pneumovax [2]. Two individuals in the adult control group had pre Typhi Vi vaccination concentrations exceeding 100 U/mL. It is possible that they had had prior contact with the pathogen or had received a previous vaccination. Ferry and colleagues identified 2/23 individuals with high concentrations Typhim Vi antibodies pre vaccination. One individual had suffered a severe systemic illness while holidaying in Asia with possible exposure to *S. typhi*. The other individual had visited the tropics several times and so it was possible they had been vaccinated in the past [6].

Robust responses were observed in both children and adult control groups and were higher in children. A strength of the present study is the inclusion of both children and adult PID patients in which the responses were significantly lower than in their respective control groups. The data from the childrens population is of particular interest clinically given the wide spread use of Prevnar in childhood vaccination schedules and the complication it may cause in interpretation of the response to Pneumovax.

Differentiation between HYPO and CVID populations is of particular importance to initiate timely, correct therapy and is recommended in guidelines [12]. Sanchez Ramon et al. reported the differentiation of those with severe antibody deficiencies from those with a milder primary immunodeficiency and healthy controls. In this study we observed similar results. CVID patients had a significantly lower response than HYPO patients and the control group with 5/8 (63%) Typhi Vi IgG concentrations lower that that obtained by any HYPO patient. The utility of using a 3 FI to discriminate CVID patients was comparable to that reported (AUC 0.83 vs 0.99) [8].

The measurement of the IgG response to Typhim Vi™ in parallel to Pneumovax™ may provide clearer understanding of T cell independent responses and potentially the diagnosis of Specific Antibody Deficiency (SAD) [1]. Schaballie and colleagues recently identified groups of individuals with both differing and similar responses to Typhim Vi™ and Pneumovax™ vaccines [13]. In the individuals with abnormal responses to both Typhim Vi™ and Pneumovax™, one patient had prolonged otorrhea [13, 14] which may be indicative of SAD.

A limitation to this study is the size of the patient groups although the data shows concordance to previous studies [6, 8]. A larger study will allow discrimination of patients with other defined PIDs based on the response to Typhi Vi vaccination.

In conclusion we demonstrate that measurement of antibodies raised in response to Typhi Vi vaccination can identify responders and non responders in both children and adult populations. In addition we show that the response to Typhi Vi can discriminate some individuals with CVID from those with HYPO. We propose that the measurement of Typhi Vi antibodies may provide an additional tool for the assessment of the response to a polysaccharide vaccine.

## Declarations

### Author contribution statement

Jeevani Kumarage: Conceived and designed the experiments; Performed the experiments.

Suranjith Seneviratne: Conceived and designed the experiments; Wrote the paper.

Vijitha Senaratne, Amitha Fernando, Kirthi Gunasekera, Bandu Gunasena: Performed the experiments.

Padmalal Gurugama: Conceived and designed the experiments.

Sudath Peiris: Conceived and designed the experiments; Contributed reagents, materials, analysis tools or data.

Stephen Harding, Antony R. Parker: Analyzed and interpreted the data; Contributed reagents, materials, analysis tools or data; Wrote the paper.

Nilhan Rajiva de Silva: Conceived and designed the experiments; Performed the experiments; Wrote the paper.

### Competing interest statement

The authors declare the following conflict of interests: Antony R. Parker and Stephen Harding are employees of the Binding Site Group Limited. The other authors declare no conflict of interests.

### Funding statement

This research did not receive any specific grant from funding agencies in the public, commercial, or not-for-profit sectors.

### Additional information

No additional information is available for this paper.
